# Involvement of Mammalian RF-Amide Peptides and Their Receptors in the Modulation of Nociception in Rodents

**DOI:** 10.3389/fendo.2014.00158

**Published:** 2014-10-02

**Authors:** Safia Ayachi, Frédéric Simonin

**Affiliations:** ^1^UMR 7242 CNRS, Laboratory of Excellence Medalis, Biotechnologie et Signalisation Cellulaire, Université de Strasbourg, Illkirch, France

**Keywords:** pain, nociception, GPCRs, RF-amide, opiates, opioid-induced hyperalgesia

## Abstract

Mammalian RF-amide peptides, which all share a conserved carboxyl-terminal Arg–Phe–NH_2_ sequence, constitute a family of five groups of neuropeptides that are encoded by five different genes. They act through five G-protein-coupled receptors and each group of peptide binds to and activates mostly one receptor: RF-amide related peptide group binds to NPFFR1, neuropeptide FF group to NPFFR2, pyroglutamylated RF-amide peptide group to QRFPR, prolactin-releasing peptide group to prolactin-releasing peptide receptor, and kisspeptin group to Kiss1R. These peptides and their receptors have been involved in the modulation of several functions including reproduction, feeding, and cardiovascular regulation. Data from the literature now provide emerging evidence that all RF-amide peptides and their receptors are also involved in the modulation of nociception. This review will present the current knowledge on the involvement in rodents of the different mammalian RF-amide peptides and their receptors in the modulation of nociception in basal and chronic pain conditions as well as their modulatory effects on the analgesic effects of opiates.

## Introduction

A tetrapeptide Phe–Met–Arg–Phe–NH_2_ (FMRF-NH_2_) has been isolated in 1977 by Price and Greenberg and was described as having cardioexcitatory properties in the neverid clam *Macrocallista nimbosa* (1). Thereafter, using specific antibodies for the COOH-terminus of this peptide, FMRF-NH_2_ immunoreactive peptides were identified in several species including mouse (2, 3) and the first two mammalian FMRF-NH_2_-like peptides, neuropeptides FF and AF, were further isolated from bovine brain (4). They represent the first members of the family of mammalian RF-amide peptides, which all share a conserved carboxy-terminal Arg–Phe–NH_2_ sequence. Additional members of this family and their receptors were identified and cloned in the late 90s and early 2000s (5). At present, five groups of mammalian RF-amide peptides have been described including neuropeptide FF (NPFF), RF-amide related peptide (RFRP), pyroglutamylated RF-amide peptide (QRFP), prolactin-releasing peptide (PrRP), and kisspeptin groups. The sequences of human and rodents RF-amide peptides, their different names as well as the name of their receptors are listed in Table 1.

**Table 1:** **Summary of nomenclature and sequences for mammalian RF-amide peptides and their receptors**.

Group	Peptide	Species	Uniprot accession number	Sequence	Receptor name
NPFF	NPFF	Human	O15130	SQ**AFLFQPQRF-NH2**	NPFFR2
		Rat	Q9WVA9	NP**AFLFQPQRF-NH2**	GPR74
		Mouse	Q9WVA8	SP**AFLFQPQRF-NH2**	HLWAR77
	NPAF (NPSF for the last eight residues)	Human	O15130	AGEGLNSQ**FWSLAAPQRF-NH2**	
		Rat	Q9WVA9	E**FWSLAAPQRF-NH2**	
		Mouse	Q9WVA8	Q**FWSLAAPQRF-NH2**	
RFRP	RFRP-1 (NPSF, GnIH)	Human	Q9HCQ7	MPHSF**ANLPLRF-NH2**	NPFFR1
		Rat	Q9ESQ9	VPHSA**ANLPLRF-NH2**	GPR147
		Mouse	Q9ESQ8		OT7T022
	RFRP-3 (NPVF)	Human	Q9HCQ7	VPN**LPQRF-NH2**	
		Rat	Q9ESQ9	ANMEAGTMSHFPS**LPQRF-NH2**	
		Mouse	Q9ESQ8	VNMEAGTRSHFPS**LPQRF-NH2**	
QRFP	QRFP43 (43RFa, P518)	Human	P83859	<EDEGSEATGFLPAAGEKTSGPLGNLAEELNG**YSRKKG GFSFRF-NH2**	QRFPR
					GPR103
		Rat	P83860	<EDSGSEATGFLPTDSEKASGPLGTLAEELSS**YSRRKGG FSFRF-NH2**	AQ27
					SP9155
		Mouse	Q8CE23	<EDGSSEAAGFLPADSEKASGPLGTLAEELSS**YSRRKGG FSFRF-NH2**	
	QRFP26 (26RFa)	Human	P83859	TSGPLGNLAEELNG**YSRKKGGFSFRF-NH2**	
		Rat	P83860	ASGPLGTLAEELSS**YSRRKGGFSFRF-NH2**	
		Mouse	Q8CE23	
PrRP	PrRP31	Human	P81277	SRTHRHSMEIRTPDINPAWYAS**RGIRPVGRF-NH2**	PrRPR
		Rat	P81278	SRAHQHSMETRTPDINPAWYTG**RGIRPVGRF-NH2**	GPR10
		Mouse	B7U2G4		hGR3
	PrRP20	Human	P81277	TPDINPAWYAS**RGIRPVGRF-NH2**	UHR-1
		Rat	P81278	TPDINPAWYTG**RGIRPVGRF-NH2**	
		Mouse	B7U2G4	
Kisspeptin	Kisspeptin-54 (metastin)	Human	Q15726	GTSLSPPPESSGSPQQPGLSAPHSRQIPAPQGAVLVQR EKDLPN**YNWNSFGLRF-NH2**	Kiss1R
					GPR54
	Kisspeptin-52	Rat	Q7TSB7	TSPCPPVENPTGHQRPPCATRSRLIPAPRGSVLVQREKDMSA **YNWNSFGLRY-NH2**	OT7T175
					AXOR12
		Mouse	Q6Y4S4	SSPCPPVEGPAGRQRPLCASRSRLIPAPRGAVLVQREKDLS T**YNWNSFGLRY-NH2**	
	Kisspeptin-10	Human	Q15726	**YNWNSFGLRF-NH2**	
		Rat	Q7TSB7	**YNWNSFGLRY-NH2**	
		Mouse	Q6Y4S4		

*Sequences in bold correspond to the conserved carboxyl-terminal sequence between human, rat and mouse peptides*.

Several RF-amide peptide receptors have been discovered: NPFFR1 (alias GPR147), NPFFR2 (alias GPR74), QRFPR (alias GPR103), prolactin-releasing peptide receptor (PrRPR) (alias GPR10), and Kiss1R (alias GPR54). They all belong to the G-protein-coupled receptor family, and share approximately 50% homology. Each receptor binds mostly one group of RF-amide peptide: NPFFR1 binds RFRP peptides, NPFFR2–NPFF group, QRFPR–QRFP group, PrRPR–PrRP group, and Kiss1R–kisspeptin group. However, *in vitro* it has been shown that NPFFR1/2 display a good affinity for all mammalian RF-amide peptides, whereas, QRFPR, PrRPR, and Kiss1R show a high level of discrimination for their endogenous peptides (6).

In this review, we will focus on the involvement of the five mammalian RF-amide peptide groups and their respective receptors in the modulation of nociception in rodents.

## NPFF, RFRP, and NPFF Receptors 1/2

Neuropeptide FF group of peptides includes NPFF and neuropeptide AF (NPAF), while RFRP group comprises RFRP-1 and -3 (also called NPSF and NPVF). NPFF and NPAF are derived from the same precursor, whereas RFRP-1 and RFRP-3 are generated from another precursor (7–10). These different peptides can all activate NPFFR1 and NPFFR2, but RFRP group displays better activity for NPFFR1, and NPFF group preferentially activates NPFFR2 (5). NPFFR1 and NPFFR2 are both coupled to G_i_ (9, 11–13). They display around 50% amino acid identity, and are encoded by two different genes concomitantly identified in 2000 by different research teams (9, 11, 12). NPFFR1 and NPFFR2 are also called OT7T022/GPR147 and HLWAR77/GPR74, respectively (see Table 1). They are expressed in all vertebrate species examined and are highly conserved, underlining their important role (14).

### NPFF, NPAF, and NPFFR2

#### Localization in the pain pathways

##### NPFF and NPAF

Neuropeptide FF and NPAF were first isolated from bovine medulla oblongata and described as having anti-opioid activity on morphine-induced analgesia in the rat (4, 15). The distribution of NPFF/NPAF peptides and mRNA has been extensively reviewed by Yang and collaborators (14). In this review, the authors pointed out that one must remain vigilant regarding the NPFF distribution by immunohistochemistry or radioimmunoassay, because the antibodies first used were against the COOH-terminus of NPFF, which is shared by other RF-amide peptides, thus results may be non-specific to NPFF. Nevertheless, NPFF presence in rodents has largely been reported in discrete central nervous system (CNS) areas by radioimmunoassay, immunohistochemistry, or *in situ* hybridization studies. The highest levels recorded were in the dorsal horn of the spinal cord and in the posterior lobe of the pituitary gland [see Ref. (14, 16)]. Fibers containing NPFF in the spinal cord seems to have intrinsic spinal origin (17). Most studies have reported the absence of NPFF mRNA or immunoreactivity in dorsal roots ganglia (DRG) except Allard et al. (18) who showed the presence of NPFF immunoreactivity in DRG, but at low level and only after blocking axonal transport [see Ref. (16)].

Neuropeptide AF (also called NPSF in rodents; see Table 1) has been detected in mouse and rat spinal cord as well as in human cerebrospinal fluid [see Ref. (14, 19–21)].

##### NPFFR2

The detailed distribution in rodents of both protein and mRNA for NPFFR2 is reviewed in Ref. (14–16, 22, 23). NPFFR2 mRNA has been detected by *in situ* hybridization and qRT-PCR in several brain regions of rodents including thalamic nuclei, hypothalamus, and superficial layers of spinal cord (10–12, 24). Using immunohistochemistry and western blot, the presence of NPFFR2 has also been detected in ventral tegmental area, hippocampus, hypothalamus olfactory tubercle, and spinal cord (25). NPFFR2 distribution in rodents has been widely described by using binding experiments (11, 26–32). The highest NPFFR2 binding sites are found in the olfactory bulb (of mice but not rat), in several thalamic nuclei, and in superficial layers of the spinal cord. However, it must be noted that, in these studies, the radiolabeled ligands used for the detection of NPFFR2 are not highly selective for this receptor and could therefore limit the relevance of the conclusions.

Like for NPFF, results concerning the presence of NPFFR2 and its mRNA in DRG or in primary afferent terminals in the spinal cord are controversial, with some studies supporting their presence and other their absence in this region [see Ref. (14–16)]. NPFFR2 mRNA has been detected in rat dorsal root ganglia and trigeminal ganglia (11). Different studies have shown a decrease of NPFFR2 binding in rats after dorsal rhizotomy, neonatal capsaicin treatment, sciatic nerve section, or spinal cord ligation (29, 33) while Lombard and collaborators did not see any decrease of binding sites after neonatal capsaicin treatment or dorsal rhizotomy suggesting that, in the rat spinal cord, NPFF receptors are mostly post-synaptically expressed (34).

Overall, the plurality of techniques leading to the same distribution pattern supports the presence of NPFFR2 in different brain structures and spinal cord and is consistent with its potential role in the modulation of nociception and sensory input.

#### Modulation of nociception

##### Effects of NPFF/NPAF on basal nociception and opiate analgesia

The modulation of nociception by NPFF/NPAF has been largely studied and reviewed in Ref. (5, 14–16, 22, 23, 35). Table 2 summarizes the effect of the different RF-amide peptides on nociception. NPFF has been described as having two different effects on pain perception depending on the site of administration. When administrated by intrathecal (i.th.) injection, NPFF showed anti-nociceptive effect, which may be considered opioid-like effect as it provoked analgesia and potentiated opioid effects. When administrated at the supra spinal level via intracerebroventricular (i.c.v.) injection, NPFF had a pro-nociceptive effect characterized by a reversal of morphine analgesia, indicating that it displays anti-opioid properties [for details, see Ref. (15)]. Furthermore, inhibition of morphine analgesia induced by i.c.v. injection of NPFF in mice was blocked when NPFF was co-administered with RF9 (either i.c.v. or subcutaneously), a selective antagonist of NPFFR1/2 (6, 36–38). RF9, also potentiated opiate analgesic effects and blocked opioid-induced hyperalgesia and analgesic tolerance both in mice and rats (38, 39). Altogether, these results support that NPFF action on nociception and opiate analgesia is mediated by NPFF receptors and that this peptide and its receptor are part of an anti-opioid system that is involved in the homeostatic control of opiates action. Hypothesis explaining NPFF anti-opioid properties are further described below. We can notice that in most reports, NPFF alone had no effect on basal nociceptive threshold but efficiently reversed morphine analgesia, suggesting that NPFF anti-opioid properties depend on opioid receptors stimulation and points to their interconnected mechanisms of action. However, it is noteworthy that in few studies, NPFF administered alone by i.c.v. lowered the nociceptive threshold measured by the tail flick test in rat, which is consistent with anti-opioid properties of this peptide (4, 40). Interestingly, in PrRPR lacking mice, NPFF administration did not reverse morphine analgesia any more, indicating that at least some NPFF actions require a functional PrRPR [see below; (41)]. Finally, NPFF-related peptides delayed the rate of acid-sensing ion channels (ASIC) desensitization causing an enhancement of acid gated currents (42), which are known to have pain modulatory properties (43). In addition, it has been demonstrated that expression of ASIC3 increased under inflammatory conditions (44). Therefore, NPFF may also modulate pain through ASICs. However, these results were obtained with high concentrations of RF-NH_2_ peptides, thus questioning the physiological relevance of these observations.

**Table 2:** **Summary of the effects of mammalian RF-amide peptides on the modulation of nociception**.

Group	Peptide	Injection	Basal nociception	Inflammatory pain	Neuropathic pain
NPFF	NPFF (or analog)	i.th.	Analgesia and potentiation of opioid effects [see Ref. (15)]	Attenuates allodynia and thermal hyperalgesia (45, 46)	Attenuates allodynia (45–47)
		i.c.v.	No effect or decreases basal nociceptive threshold and reverses morphine analgesia [see Ref. (15)]		Attenuates tactile allodynia (46, 47)
		Intraperitoneal		Attenuates flinching behavior in formalin test (48)	Decreases mechanical hypersensitivity (48)
	NPAF	i.th.	Potentiates morphine analgesia and reverses morphine analgesic tolerance (49)		
		i.c.v.	Increases/decreases morphine analgesia (19, 50)		
RFRP	RFRP-1 (NPSF, GnIH)	i.th.			Thermal anti-nociception and tactile anti-allodynia (51)
		i.c.v.	Decreases morphine analgesia (10)	Decreases morphine analgesia (10)	
	RFRP-3 (NPVF)	i.c.v.	Decreases thermal nociceptive threshold (6); potentiates/decreases/no effect on morphine analgesia (6, 37, 50)		
QRFP	QRFP26 (26RFa)	i.th.	No effect on mechanical or thermal nociception (52, 53)	Decreases mechanical allodynia; inhibits agitation behavior in formalin test (52, 54)	Antiallodynic effect (53)
		i.c.v.	No effect on thermal nociception (54); decreases thermal nociceptive threshold (6)	Inhibits agitation behavior in formalin test (52, 54)	
PrRP	PrRP20	i.th.	No effect (55)		
		Intracerebral	Analgesia [nucleus tractus solitary; (55)]		Attenuates tactile allodynia (periaqueductal gray, nucleus tractus solitarius) or no effect [caudal ventrolateral medulla; (55)]
		i.c.v.	Hyperalgesia (6, 41, 55)		
Kisspeptin	kisspeptin-54	Intraplantar	Nocifensive response; decreases thermal pain threshold (56)		
	kisspeptin-10	i.c.v.	Hyperalgesia and anti-morphine activity (6)		

Neuropeptide AF has been shown to display NPFF-like bioactivity. NPAF injection, in the lateral ventricle of mice, increased or decreased morphine-induced analgesia in the tail flick test depending on NPAF and morphine amounts used (50) while another study showed that i.c.v. administration of NPAF in mice potently reversed morphine-induced analgesia in the tail flick test (19). I.th. administration of a low dose of NPAF in the rat potentiated morphine anti-nociception in tail flick and paw-pressure tests and efficiently reversed morphine analgesic tolerance (49). Altogether, these results demonstrate NPAF implication in opioid-modulating system. Like NPFF, NPAF can increase the amplitude of the sustained current of ASIC (57) indicating its eventual role in neuron excitability and then in nociception. However, in ASIC3 knockout mice, nociceptive behavior induced by NPAF subcutaneous injection is similar than in wild type animals, suggesting that ASIC3 is not involved in this effect (58).

##### Anti-opioid properties

Tolerance is defined by the loss of efficacy of a given compound after prolonged treatment, which leads to the necessity to increase the dose to reach the same effect. Two different hypotheses have been proposed to explain tolerance to morphine. One is based on different molecular mechanisms including functional selective desensitization of receptor signaling, receptor endocytosis, and degradation (59–63). The second one proposes the existence of a homeostatic equilibrium between the anti-nociceptive opioid system and pro-nociceptive anti-opioid systems (64, 65). In this model, activation of the opioid system by an exogenous opiate produces analgesia, but also stimulates the release of endogenous anti-opioid molecules, which produces a pro-nociceptive effect. This pro-nociceptive effect ramps up during chronic administration of the opiate and thus opposes to its analgesic effect. It is then necessary to increase opiate doses to overcome the activation of anti-opioid systems and produce an analgesic effect, thus explaining the tolerance described following chronic opioid treatment. Moreover, upon cessation of the opiate treatment, the opioid system is no longer activated while the anti-opioid system remains activated by endogenous anti-opioid molecules, which explains the decrease in the basal nociceptive threshold or hyperalgesia observed following disruption of chronic morphine treatment. This phenomenon is called opioid-induced hyperalgesia.

Neuropeptide FF has been shown to participate in the adaptive processes that counteract the opioid effects or change in the nociceptive threshold leading to opioid-induced hyperalgesia and tolerance. Indeed, increase in NPFF-like immunoreactivity has been observed in the brain and spinal cord during chronic or acute morphine treatment (66–68), supporting the key role of NPFF in these processes [see Ref. (35, 64)]. It has also been shown that reducing NPFF expression by central administration of antibodies or antisense oligonucleotides attenuated the tolerance to morphine analgesia (69, 70). Moreover, when NPFFR1/2 were pharmacologically blocked in rats or mice with RF9, there was a potentiation of opiate analgesic effects, a prevention of the development of opiate tolerance and an abolition of hyperalgesia induced by acute or chronic administration of different opiates including, fentanyl, heroin, and morphine (38, 39). Furthermore, triple knockout mice for μ-, δ-, κ-opioids receptors (corresponding to MOR, DOR, and KOR, respectively) are hyperalgesic, which supports the idea that MOR/DOR/KOR deletion certainly affects the balance between opioid and anti-opioid systems, and then, may lead to an increase in NPFF system efficacy. Accordingly, MOR/DOR/KOR triple knockout mice showed an increase in NPFFR2 binding sites in several brain region and spinal cord (71). Finally, it has been shown that chronic i.c.v. infusion of NPFF downregulated MOR binding sites in the rat brain (72), whereas injection of antiserum against NPFF (anti-NPFF IgG) upregulated MOR binding sites (73). This suggests that density of MOR is regulated by NPFF, raising another possible way to induce anti-opioid effects.

Regarding morphine withdrawal and rewarding effects, NPFF also shows anti-opioid properties. NPFF i.c.v. injection produced an abstinence syndrome in morphine-dependent rats (66), and IgG i.c.v. injection from an antiserum against NPFF attenuated naloxone-induced withdrawal syndrome (70, 74), suggesting that NPFF modulates the different effects of opioids. I.c.v. injection of NPFF analog (dNPA) in mice counteracted the c-Fos expression induced by morphine in the shell of nucleus accumbens (75) known to be required for the acquisition of morphine-conditioned place preference (76). NPFFR1/R2 blockade with RF9 increased morphine-induced conditioned place preference and decreased naltrexone-precipitated withdrawal syndrome (38). Accordingly, injection of the NPFF analog (1DMe)-NPYF inhibited the rewarding effect of morphine (77), and acute administration of NPFF inhibited morphine-induced hyperlocomotion (78).

Overall, these results suggest that NPFF/NPFFRs system opposes to opioid effects, highlighting its role as an anti-opioid system.

##### Cellular effect

Hypothesis to explain the activation of NPFF/NPFFRs anti-opioid system, by the stimulation of the opioid system, may be that the neuronal circuitry implicated in both systems is interconnected, or it may exist a crosstalk between receptors in the same neurons involving a correlation/crosscheck in the cellular effects (79). In agreement with this second hypothesis, it has been shown that in rat spinal ganglion neurons the NPFF analog (1DMe)-NPYF reversed the inhibition by DAMGO of depolarization-evoked rise in intracellular Ca^2+^ (80), suggesting that NPFF could block the inhibition of morphine analgesia by reversing the effect of mu agonists on Ca^2+^channels. Another possibility to explain anti-opioid activity of NPFFR2 receptors is based on physical interaction between NPFFR2 and MOR. In fact, it has been shown that NPFF agonist promoted a heteromeric association between NPFFFR2 and MOR that changed the lateral diffusion of MOR, which seems to move MOR away from its signaling partners, thus reducing response to opioids (81). Moreover, NPFF have been shown to modify the G-protein environment of MOR, which may also participate in the mechanism by which this peptide reduces the inhibitory activity of opioids (82). More generally, stimulation of NPFF receptor seems to provoke changes in MOR-associated signaling that could occur by receptor heteromerization and/or alteration in signaling transduction pathways [see Ref. (35)].

Concerning the pro-opioid properties, Mollereau et al. have shown that, similarly to opioid agonists, NPFF increased voltage-dependent potassium outward currents in F-11 DRG cell line (hybridoma derived from rat DRG and mouse neuroblastoma), which may explain the similar anti-nociceptive actions of NPFF and opioid agonists at the spinal level (83). Regarding the opioid potentiating effect of intrathecally injected NPFF, it has been proposed that activation of NPFF receptors could result in a functional blockade of δ opioid autoreceptors in the spinal cord, which are involved in an inhibitory feedback on Met-enkephalin release (84). However, the molecular mechanisms involved in this effect are still unclear and this hypothesis is not supported by studies in Chinese hamster ovary cell line showing that NPFF analog (1DMe)-NPYF had no intrinsic activity but enhanced DOR-mediated inhibition of forskolin-stimulated cAMP accumulation and phosphorylation of ERK2 (85).

##### Involvement of NPFF system in persistent pain

Neuropeptide FF and NPFFR2 seem to be implicated in inflammatory pain. Indeed, several studies, using different models of inflammatory pain, have shown that inflammation-induced modulation of NPFF or NPFFR2 at the mRNA and protein levels [see Table 3 in Ref. (14)]. For instance, persistent pain induced by carrageenan inflammation upregulated NPFF and NPFFR2 gene expressions in the rat spinal cord, suggesting an involvement of spinal NPFF system in inflammatory pain (8, 24, 86). In the rat, it has also been shown that during acute colonic inflammation there is an up-regulation of supraspinal NPFFR2 (86). Moreover, in the spinal cord of rats with tibio-tarsal joint inflammation (induced by *Mycobacterium butyricum* in Freund’s adjuvant injected into the tibio-tarsal joint), there is an increase of [^125^I]-1DMe-NPYF binding (87) while carrageenan induced an increase of NPFF immunoreactive cell bodies in spinal cord (88) and an increase of NPFFR2 immunoreactivity in primary afferent terminals (24). Otherwise, it has been shown that NPFF administration (i.th. or i.c.v.) attenuated allodynia induced by injection of Freund’s Complete Adjuvant in the rat paw (46) while (1DMe)-NPYF i.th. administration decreased mechanical allodynia and thermal hyperalgesia induced by carrageenan inflammation (45). More recently, another study showed that an intraperitoneal injection of the NPFFR2 agonist AC-263093 attenuated flinching behavior in formalin test, and thermal hyperalgesia induced by carrageenan injection in hotplate test (48). Very recently, an *in vitro* study using LPS-stimulated macrophages has shown that NPFF suppressed the production of nitric oxide, an important signal transmitter during inflammatory processes, suggesting a possible anti-inflammatory action of this peptide (89). This effect was blocked by a pretreatment with RF9, indicating that it is mediated by NPFF receptors. Finally, in mice with chronic inflammatory pain induced by complete Freund’s adjuvant, the decrease of morphine rewarding properties were associated with an increase of NPFFR2 agonist [^125^I]-EYWSLAAPQRF-NH_2_ binding in several brain regions involved in morphine reward including the shell of the nucleus accumbens, the major islands of Calleja and the ventral endopiriform nucleus. Altogether, these data indicate that NPFF and NPFFR2 are probably upregulated in different models of inflammatory pain in rodents and that NPFF displays antiallodynic and anti-hyperalgesic effects in these models.

In rats with neuropathic pain, NPFF mRNA was not upregulated neither in the spinal cord nor in the brainstem, while NPFFR2 mRNA was slightly upregulated only in the brainstem (8, 86). NPFF administrations either i.c.v., i.th. or directly into the periaqueductal gray attenuated tactile allodynia induced by chronic neuropathy in rats (46, 47). Moreover, i.th. administration of the NPFF analog (1DMe)-NPYF decreased cold and mechanical allodynia observed in rats with spinal nerve ligation (45) and intraperitoneal injection of NPFFR2 agonist AC-263093 attenuated mechanical hypersensitivity in the same model of neuropathy (48). In summary, although NPFF and NPFFR2 mRNA do not seem to be strongly regulated in neuropathic pain models, NPFF and NPFF-related compounds display antiallodynic properties in neuropathic animals.

In a model of cancerous pain induced by melanoma cells injected in the hind paw of the mouse, the binding of NPFFR2 agonist [^125^I]-EYWSLAAPQRF-NH_2_ increased in several brain areas involved in the rewarding properties of opiates, similarly to what was observed in a model of chronic inflammation (see above). This increase of binding was associated with a decrease of morphine’s motivational properties tested with the place preference paradigm. These data suggest that NPFF contributes to the suppression of morphine rewarding effects in this model of cancerous pain (90).

Overall, NPFF and NPFF-related compounds have been shown to display antiallodynic/hyperalgesic properties in different models of chronic pain, which contrast with the pro-nociceptive anti-opioid properties of NPFF that have been observed in animals that were chronically treated with opiates.

### RFRP-1, RFRP-3, and NPFFR1

The gene encoding RFRP-1 and RFRP-3 have been identified and cloned in the beginning of 2000s by two research teams through databases searches (9, 10). These two different peptides, also known as NPSF and NPVF, derived from the same precursor protein and constitute the RFRP group [(9, 10); see Table 1]. RFRP-1 seems to be the mammalian homolog of LPLRF-amide, a peptide isolated in 1983 in avian brain (3) and of gonadotropin-inhibitory hormone (GnIH) characterized from quail brain (91). RFRP-1 and RFRP-3 activate preferentially NPFFR1, which triggers the G_i/o_ signal transduction pathway (9, 11).

#### Localization in the pain pathways

##### RFRP-1 and RFRP-3

Neurons containing RFRP-1 and RFRP-3 proteins and RFRP mRNA have been found by using immunohistochemical analyses and *in situ* hybridization in rat and mouse CNS (9, 10, 92–96). Their highest level has been found in the hypothalamus (periventricular and ventromedial nucleus), and fibers containing RFRP-1 and RFRP-3 are widely distributed in the brain (94). These results were confirmed very recently by the analysis of transgenic rats expressing an enhanced green fluorescent protein tagged to the RFRP promoter (97). Neurons containing RFRP immunoreactivity and mRNA are distinct from those containing NPFF (94). In mice, RFRP-immunoreactive fibers were found in the superficial layer of spinal trigeminal nucleus and dorsal horn of the spinal cord (93), while in rats, low levels of RFRP-1 immunoreactivity and no mRNA were detected in these regions (51, 94). From these observations, Pertovaara et al. (51) suggested that RFRP neurons localized in rat hypothalamus may contribute to well-known descending pain modulatory pathways (98), which project to nucleus of the solitary tract and spinal cord.

##### NPFFR1

NPFFR1 transcript was identified both in RT-qPCR and *in situ* hybridization experiments in several structures of the rat CNS. Highest signals were detected in the lateral septum and in various hypothalamic and thalamic nuclei (9–11). Accordingly, a high level of NPFFR1 binding sites was found in these regions both in the rat and mouse (30–32). NPFFR1 mRNA and binding sites were weakly or not detectable in the superficial layers of the spinal cord, depending on the strains of rodents used (10, 11, 31). Overall, there is a good correlation between the distribution of NPFFR1 receptor and its endogenous ligands RFRP-1/-3.

#### Modulation of nociception

Modulatory effects of both RFRP-1 and RFRP-3 on nociception have been reported (see Table 2). I.c.v. injection of RFRP-1, when given alone, had no effect on hot plate latencies in the rat but when co-administrated with morphine, it decreased or blocked morphine analgesia in hot plate and formalin tests (10). These data suggest that, like NPFF, RFRP-1 action depends on opioid receptors activation, supporting its role in an anti-opioid system related to the opioid system. In neuropathic rats, RFRP-1 produced pain suppressive effects depending on the modality of the noxious test and the localization of the injection (51). Indeed, i.th. injection of RFRP-1 induced thermal anti-nociception (tail flick test) and tactile anti-allodynia (Von Frey filament), while injection in the solitary tract nucleus produced only mechanical anti-hyperalgesia (paw-pressure test). Furthermore, tactile anti-allodynia produced by RFRP-1 was attenuated by naloxone, indicating that opioid receptors are involved in the spinal action of RFRP-1.

In mice, i.c.v. injection of VPNLPQRF-NH_2_ (human RFRP-3) in the lateral ventricle has been reported to either potentiate morphine analgesia at high doses [10–32 nmol; (50)] or decrease morphine analgesia at similar doses [20 nmol; (37)]. Conversely, i.c.v. injection in mice of RFRP-3 from mouse origin (10 nmol) had no effect on morphine analgesia but significantly decreased thermal nociceptive threshold when injected alone and this effect was reversed by RF9 (6). In SH-SY5Y cells transfected with NPFFR1 and endogenously expressing mu and delta opioid receptors, RFRP-3 acted like opioids by inhibiting adenylyl cyclase activity and voltage-gated Ca^2+^ currents, whereas preincubation of those cells with RFRP-3 induced an anti-opioid activity characterized by a decrease of response to opioids on calcium signaling (99). Finally, NPFFR1 was shown to be downregulated in several brain regions of triple MOR/DOR/KOR knockout mice, suggesting an interaction between opioid and RFRP/NPFFR1 systems (71).

Altogether, these results indicate that RFRP-1/3 play a role in the modulation of nociception and opioid effects, presumably via their receptor NPFFR1. However, if we consider that no NPFFR1 has been found in the spinal cord of rodents and that RFRP-1/-3 peptides can bind to NPFFR2 subtype, this receptor may be involved in the spinal nociceptive modulating effect of these peptides (6). Moreover, RFRPs as well as NPFF and NPAF have also been shown to bind and activate some Mrgs (mas-related genes), which are G-protein-coupled receptor expressed in DRG by a specific subset of sensory neurons (100, 101), suggesting that these receptors could also represent relevant targets for nociceptive effects of these peptides.

## QRFP and QRFP Receptor

Pyroglutamylated RF-amide peptide and its receptor QRFPR are the most recently discovered mammalian RF-amide peptide and receptor, respectively (102–104). QRFPR is also known as GPR103, AQ27, SP9155, and QRFP as P518, 26RFa/43RFa (see Table 1). 43RFa is a NH_2_-terminally elongated form of 26RFa. Both peptides arise from the same precursor and were isolated from human hypothalamus and spinal cord, while only 43RFa was isolated from rat brain (102, 105). The primary sequences of human and rodent 26RFa display more than 80% identity (102). The nomenclature QRFP26 and QRFP43 will be used throughout the text for 26 and 43RFa, respectively. QRFPR have been shown to activate the G_q_/G_i_ signal transduction pathways in heterologous cells (103, 104), while QRFP26 has been shown to stimulate cAMP production by rat pituitary cells (102). In rodents, two receptor subtypes have been identified QRFPR1 and QRFPR2 (105, 106). Human QRFPR shares 83/84 and 79/82% amino acid identity with mouse and rat QRFPR1 and QRFPR2, respectively, while mouse QRFPR1 and R2 share with each other 75% amino acid identity (105). QRFP26 and 43 have also been shown to display high affinity for and agonist activity at both NPFF receptor subtypes (6, 107).

### Localization in the pain pathways

Pyroglutamylated RF-amide peptide mRNA distribution has been studied in detail in mouse and rat CNS by *in situ* hybridization (102, 103, 105, 106). QRFP transcript was detected almost exclusively in the hypothalamus including ventromedial hypothalamic nucleus, the lateral hypothalamic area, the arcuate nucleus, the retrochiasmatic area, and periventricular nucleus [see Ref. (108)]. In mouse, low levels of QRFP transcript were identified by RT-qPCR in spinal cord (105). In naive rat, QRFP-like immunoreactivity was observed in the L5 DRG (52, 53) where it was mainly located in small to medium sized neurons suggesting a relationship with C-fibers and therefore nociceptive transmission (53). Similar observations were made after a partial spinal nerve ligation (53).

Conversely to QRFP transcript, QRFPR1 and R2 mRNAs are widely distributed in the CNS of rodents including spinal cord [see Ref. (108)]. High expression levels were observed in several different hypothalamic and thalamic nuclei as well as brainstem nuclei including dorsal raphe nucleus and the locus coeruleus that are critical regions involved in pain control. In mice, the mRNA distribution of QRFPR1 and R2 displayed no overlap with each other (105). However, the transcript for both receptor subtypes has been detected in the spinal cord (105, 109). Like QRFPR transcripts, QRFP binding sites are widely distributed in the brain and the spinal cord with a high density in the superficial layers of the dorsal horn [see Ref. (108)]. QRFP binding sites have been described in nuclei involved in processing of pain, such as the parafascicular thalamic nucleus, the locus coeruleus, the dorsal raphe nucleus, the parabrachial nucleus. However, in the rat, QRFPR mRNA-containing cells are particularly expressed in the midbrain, the pons, and the medulla oblongata, while QRFP binding sites are widely distributed throughout the brain and the spinal cord. These differences may be explained by the fact that QRFP also bind to NPFFR2 (110). QRFPR-like immunoreactivity was observed in the superficial lamina of the spinal dorsal horn but not in DRG (52). Taken together, these data suggest the involvement of QRFP-QRFPR system in the nociceptive transmission and/or integration.

### Modulation of nociception

As summarized in Table 2, in naïve rats, i.th. injection of QRFP26 had no effect neither on mechanical nor on thermal nociception [tested with the hotplate test and Von Frey filaments, respectively; (52, 53)], and i.c.v. injections had no effect on thermal nociception (54). However, a recent study showed that i.c.v. administration of QRFP26 in mice provoked a decrease of thermal nociceptive threshold (6). This discrepancy could be explained by several differences between these studies including the species (rat versus mouse) and nociceptive test used (hot plate versus tail immersion) as well as amount of QRFP injected (three or four times higher in the rat study than in the mouse study).

The effect of QRFP26 has also been examined in different models of persistent pain in rats including inflammatory pain (carrageenan and formalin tests) and neuropathic pain (partial sciatic nerve ligation). Concerning inflammatory pain, it has been shown that i.th. injection of QRFP26 attenuated the level of mechanical allodynia induced by paw carrageenan injection, while i.th. or i.c.v. injections of this peptide inhibited the phase 1 and phase 2 of the agitation behavior induced by paw formalin injection (52, 54). Similarly, i.th. and i.c.v. injections of QRFP produced an antiallodynic effect in a model of partial sciatic nerve ligation (53).

These data together with the observations made on naïve animals in the same studies suggest that QRFP modulates nociceptive transmission only during inflammation or neuropathy and has no effect on physiological nociceptive transmission. In addition, Yamamoto et al. pointed out that i.th. but not i.c.v. injection of QRFP suppressed the expression of Fos-like immunoreactivity induced by paw formalin injection in the superficial layers of the spinal dorsal horn, suggesting thus, an implication of QRFP in spinal sensitization induced by paw formalin, carrageenan injection, or partial spinal nerve ligation (52).

Recently, a novel QRFP peptide named TC26RFa has been identified from Chinese tree shrews (111). I.p. injection of TC26RFa in mice induced analgesia in thermal nociceptive tests (tail flick and hot plate tests), in a model of visceral pain, and in the formalin test, and stimulated secretion of anti-inflammatory factors from RAW 264.7 cells (111). In the future, it will be important to determine whether these effects occur through the direct activation of mouse QRFPR and which receptor subtype is involved in these effects.

## PrRP and PrRP Receptor

Prolactin-releasing peptide, named as such for its prolactin-releasing activity, has been first isolated by Hinuma and collaborators as stimulator of arachidonic acid release from cells expressing an orphan G-protein-coupled receptor cloned from human pituitary and called hGR3 (112). This receptor is nearly identical to a previously cloned orphan receptor called GPR10 (113) and its rat ortholog was cloned from hypothalamus and called UHR-1 (114). Its official name is presently prolactin-releasing peptide receptor (PrRPR). Both short (PrRP20) and NH_2_-terminally elongated (PrRP31) forms of PrRP originate from the same precursor protein of 98 amino acids [see Table 1 and Ref. (115)]. Several results suggest that PrRPR is coupled to either G_q_ or G_i/o_. Indeed, in CHO cells expressing PrRPR, PrRP induced intracellular Ca^2+^ influx and a decrease of forskolin-stimulated intracellular cAMP production (112). It has also been shown that PrRP, in rat pituitary tumor cells, stimulated the activation of ERK that was blocked by inactivating G_i/o_ by pertussis toxin (116) while in HEK293 cells, PrRPR stimulation induced intracellular Ca^2+^ influx but had no effect on cAMP production (117). Interestingly, Lin et al. have shown that PrRPR can associate with PSD-95/Disks-large/ZO-1 (PDZ) domain proteins (118). PDZ domain proteins are involved in mechanisms of scaffolding and targeting of proteins to specific subcellular domains [see Ref. (119)]. It has also been shown that PDZ domain proteins can interact with AMPA receptors, suggesting that PrRPR may modulate neurotransmission at glutamatergic synapses (118). Both PrRP20 and 31 bind with very high affinity to PrRPR, but they have also been described to display high affinity for and agonist activity at both NPFFR1 and NPFFR2 (6, 107, 120).

### Localization in the pain pathways

Prolactin-releasing peptide and PrRPR are widely distributed in the nervous system. Their detailed distribution in rodent CNS has been recently reviewed [see Ref. (23, 115, 121)]. In the rat (and human) PrRP mRNA is expressed in several regions that have been involved in the processing of nociceptive inputs including the medulla oblongata and the nucleus of solitary tract as well as dorsomedial hypothalamic nucleus. PrRP-immunoreactive fibers are present in many brain regions including several areas important for pain control including ventrolateral medulla, basolateral amygdaloid nucleus, and bed nucleus of the stria terminalis. No PrRP has been detected in the spinal cord using immunohistochemistry or *in situ* hybridization (55).

Prolactin-releasing peptide receptor mRNA is broadly expressed in the brain and the highest expression is in the anterior lobe of the pituitary (122). PrRPR mRNA has also been shown to be highly expressed in lateral and medial parabrachial nuclei, which are key region involved in the central processing of nociceptive inputs from spinal lamina I–II and spinal trigeminal cells (123). Autoradiographic binding experiments with [^125^I]-PrRP31 only identified a strong signal in the reticular nucleus of the thalamus as well as light binding in the periventricular nucleus (124).

Overall, there is a good correlation between the localization of PrRP fibers and its receptor. However, there are some discrepancies that suggest that PrRPR might not be the only receptor for PrRP. As *in vitro* binding experiments have shown that PrRP also display high affinity for NPFFR1 and NPFFR2 (6, 107, 120), it is tempting to speculate that these receptors could also represent endogenous targets of PrRP in some brain areas. In agreement with this hypothesis, we have shown that in rats the increase in blood pressure and heart rate induced by i.c.v. administration of PrRP is blocked by the NPFFR1/R2 selective antagonist RF9, while they are still present in Otsuka Long-Evans Tokushima Fatty (OLETF) rat strain, in which the GRP10 receptor gene was mutated (125).

### Modulation of nociception

Prolactin-releasing peptide modulates nociception in various ways (see Table 2). Indeed, when administered in rats by i.th. injection, PrRP20 produced no significant anti-nociception, while intracerebral injection of PrRP20 in the nucleus tractus solitarius provoked a strong analgesia in tail flick and paw-pressure tests (55). Conversely, PrRP induced significant hyperalgesia in tail immersion test when administered i.c.v. in mice (6, 41) and provoked a weak hyperalgesia in paw-pressure test when injected in the rat caudal ventrolateral medulla (55), a structure known to be involved in descending control of pain (126–128). These data indicate that PrRP modulates nociception in rats and mice under basal condition and nociceptive or anti-nociceptive actions of this peptide depend on the site of injection.

It has also been shown that PrRP plays a role in the modulation of nociception under neuropathic conditions. Indeed, in rats with spinal nerve ligation, PrRP attenuated tactile allodynia when it was injected in the periaqueductal gray or in the nucleus tractus solitarius, but had no effect when administered in the caudal ventrolateral medulla (55).

Based on the hypothesis that PrRPR participates to an anti-opioid system, Laurent et al. studied the consequence of PrRPR deletion on opiate response (41). They showed that, compared to wild type, PrRPR-knockout mice have higher nociceptive thresholds and stronger stress-induced analgesia, and these differences are suppressed by naloxone. Moreover, the hyperalgesic effect of i.c.v. administration of PrRP as well as its capacity to block morphine analgesia was absent in PrRPR-knockout mice. These animals also displayed a potentiation of morphine induced analgesia, a reduction of morphine tolerance, an enhancement of acquisition of morphine-induced conditioned place preference and a decrease of the severity of naloxone precipitated morphine withdrawal syndrome. These results suggest that PrRP–PrRPR system participate in an anti-opioid system (41). More recently, we have shown that the hyperalgesic and anti-morphine effects of PrRP in wild type mice were blocked by the selective NPFFR1/R2 antagonist RF9, indicating that NPFF receptors are also critically involved in these effects. Altogether, these data show that PrRPR and NPFF receptors are both mandatory for the action of PrRP on nociception. Similarly, these receptors seem both essential for the action of NPFF since its anti-morphine effect was absent in PrRPR-knockout animals (41) and was blocked in wild-type mice by RF9 (6).

Overall, these data indicate the existence of a functional interaction between NPFFR1/2 and PrRPR that is critical for the effect of NPFF and PrRP on nociception. Whether this interaction occurs through direct heterodimerization of these receptors or whether it is due to expression of both receptors in different cells from the same circuit remains to be investigated.

## Kisspeptin and Kiss1 Receptor

Kisspeptin is a 54-amino acid peptide first discovered as a metastasis suppressor, hence was also named metastin (129). In the human, Kiss1 gene codes for a 145-amino acid precursor protein, which is cleaved to the 54-amino acid protein (kisspeptin-54) or into shorter products (kisspeptin-10, -13, -14) that all share the same COOH-terminal Arg–Phe–NH_2_ sequence (130). In rodents, the largest proteolytic product of the kisspeptin precursor is composed of 52 amino acids (kisspeptin-52), and the RF-amide sequence is substituted by an Arg–Tyr–NH_2_ motif (see Table 1). Kisspeptin activates Kiss1R (also named GPR54, AXOR12, or OT7T175). This receptor has first been cloned from the rat brain (131) and was deorphanized in 2001 by three independent research groups (130, 132, 133). GPR54 is known to act via G_q_/G_11_ signal transduction pathways [see Ref. (134)]. Kisspeptin has also been described as capable to activate different set of interconnected signals in a cell type-dependent manner [see Ref. (135–138)]. It has also been shown that kisspeptin-10, -13, and -54 display high affinity binding and activate both NPFF receptor subtypes (6, 139, 140). Although Kisspeptin was originally identified as a metastasis suppressor, this peptide and its receptor Kiss1R are now largely recognized to have a central effect on neuroendocrine regulation of reproduction. This aspect has recently been reviewed in details by Pinilla and collaborators (141).

### Localization in the pain pathways

In the mouse and rat brain, kisspeptin transcript and protein have been detected in several hypothalamic nuclei that are involved in the control of gonadotropin secretion [see Ref. (142)]. Outside of hypothalamus, kisseptin-immunoreactive fibers were identified in relatively few regions of mouse brain, some of which being critically involved in the control of nociception including the paraventricular thalamic nucleus, periaqueductal gray, and locus coeruleus (143). In this study, the antisera specificity was controlled with brains of Kiss1 knockout mice thus insuring that the immunoreactivity observed is due to kisspeptin and not other RF-amide peptides. In the rat, kisspeptin mRNA and immunoreactivity have been found in the dorsal horn of the spinal cord and in L4/L5 DRG (144, 145). In DRG, kisspeptin is expressed in large-sized neurons and also in unmyelinated small- and medium-sized neurons, which are considered like nociceptors (145).

Kiss1 receptor mRNA has been detected in several regions of rodents’ brain [see Ref. (142)]. In the mouse, by using X gal histochemistry in a transgenic Kiss1RLacZ knock-in mouse, Herbison et al. identified Kiss1R positive cells mainly in hypothalamus and hippocampus, and in several discrete brain regions such as periaqueductal gray and cuneate nucleus of brainstem, which are involved in pain control (146). Kiss1 immunostaining have been found in rat DRG neurons and in lamina I and II of the dorsal horn of the spinal cord. In DRG, Kiss1R distribution is similar to that of kisspeptin (145). Kiss1R has also been found, by immunohistochemical analysis, in peripheral nerve endings of PGP9.5-positive sensory fibers in the mouse skin (56).

All these data suggest that kisspeptin/Kiss1R system may play a role in the modulation of nociception.

### Modulation of nociception

There are few reports that describe the effect of kisspeptin on nociception in rodents (see Table 2). In naïve mice, intraplantar injection of kisspeptin induced a small nocifensive response and a decrease of thermal pain threshold in the hot plate test (56) while i.c.v. administration of kisspeptin-10 induced hyperalgesia and anti-morphine activity (6). However, these latter effects were blocked by the NPFF1R/2R antagonist RF9 indicating that these receptors are important for the effects of kisspeptin-10 on nociception. If we consider that kisspeptins display high affinity for Kiss1R and NPFFR1/R2, the receptor(s) that constitutes the direct target of kisspeptins in these effects still remains to clarify.

In addition to its involvement in basal nociception, i.th. and intraplantar injections of kisspeptin have been shown to provoke hyperalgesia in the first and second phases of the formalin test and to enhance TRPV1 phosphorylation at Ser800 at the site of injection and ERK1/2 phosphorylation in the dorsal horn (56). Moreover, in formalin test, the blockade of the endogenous action of kisspeptin using an antagonist of Kiss1R, p234 (intraplantar and i.th.), caused analgesia. From these results, Spampinato et al. proposed that kisspeptin, by activating Kiss1R, may lead to peripheral nociceptive sensitization through the modulation of TRPV1 phosphorylation as well as to central sensitization via the activation of the ERK/MAPK pathway in the dorsal horn of the spinal cord (56). Finally, in a model of chronic inflammatory pain (complete Freund adjuvant), kisspeptin and Kiss1R transcripts and proteins were upregulated in DRG and dorsal horn neurons (145).

Altogether, these data attest that kisspeptin regulates pain and/or nociceptive sensitization in mice, and may be predominantly implicated during inflammatory pain.

## Conclusion

Mammalian RF-amide peptides and their receptors have been implicated in the modulation of several functions including feeding, reproduction, and cardiovascular regulation. Data from the literature, including tissue distribution and *in vivo* activity of RF-amide peptides, now provide emerging evidence that, not only NPFF/NPFFR2 system but all RF-amide peptides and their receptors are involved in the modulation of nociception, either in basal or chronic pain conditions. Moreover, several of these peptides and their receptors seem to participate to an anti-opioid pro-nociceptive system that controls anti-nociceptive and possibly other functions of opiates. Future studies, by using genetic and selective pharmacological tools, will be necessary to clearly delineate the role of each of these systems in the modulation of nociceptive inputs. Ultimately, these studies should lead to the identification of novel targets for pain treatment and for improving the efficacy of opiates and limit the development of tolerance following chronic treatment.

## Conflict of Interest Statement

The authors declare that the research was conducted in the absence of any commercial or financial relationships that could be construed as a potential conflict of interest.

## Abbreviations

ASIC, acid-sensing ion channels; CNS, central nervous system; DOR, δ-opioid receptor; DRG, dorsal root ganglia; i.c.v., intracerebroventricular; i.th., intrathecal; KOR, κ-opioid receptor; MOR, μ-opioid receptor; NPAF, neuropeptide AF; NPFF, neuropeptide FF; PrRP, prolactin-releasing peptide; QRFP, pyroglutamylated RF-amide peptide; RFRP, RF-amide related peptide.
